# Supplementary material for: Oxidative Cyclization at ortho-Position of Phenol: Improved Total Synthesis of 3-(Phenethylamino)demethyl(oxy)aaptamine
Source: Mar Drugs. 2023 May 19;21(5):311. doi: 10.3390/md21050311 (PMC10221624; doi:10.3390/md21050311)
Supplement: Supplementary file 1 [file marinedrugs-21-00311-s001.zip › marinedrugs-2398187-supplementary.pdf]

Supporting Information

Oxidative Cyclization at *ortho*-Position of Phenol: Improved Total synthesis of 3-(phenethylamino)demethyl(oxy)aaptamine

Yuki Nakatani, Risa Kimura, Tomoyo Kimata and Naoyuki Kotoku *

College of Pharmaceutical Sciences, Ritsumeikan University, 1-1-1 Noji-higashi, Kusatsu 525-8577, Shiga, Japan

Contents

^{1}H- and ^{13}C-NMR spectra for new compounds: pages S2-S14

^{1}H NMR (500 MHz, $CDCl_3$)

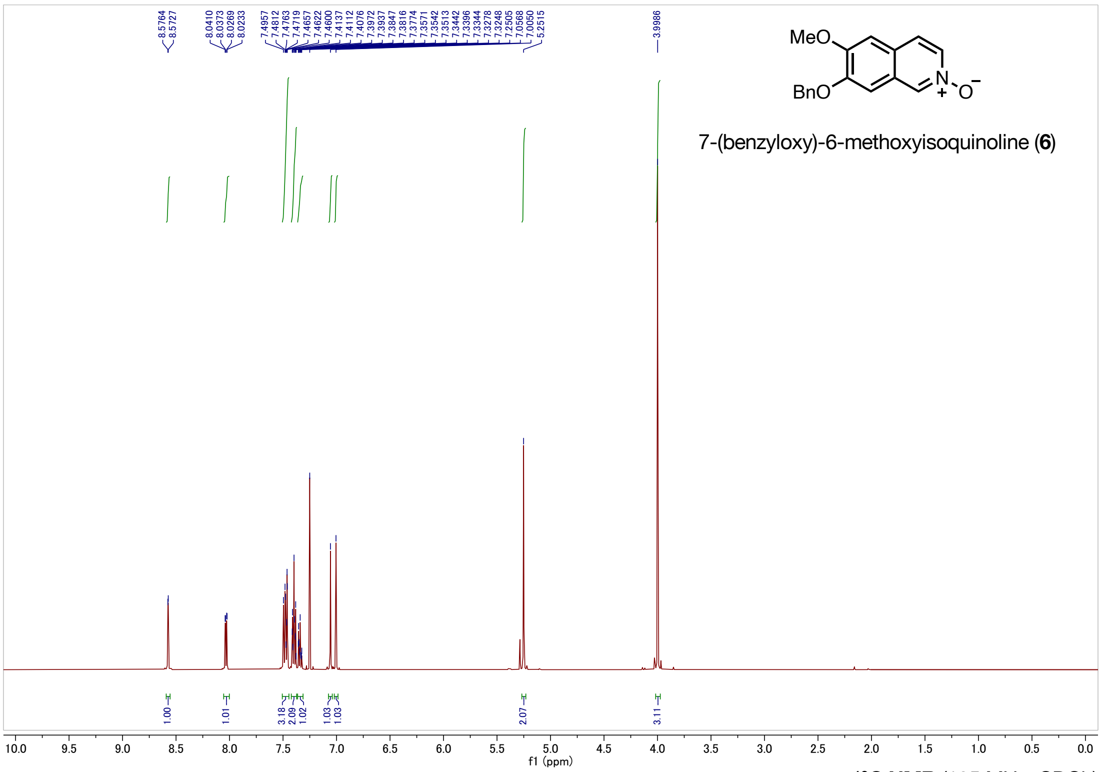

7-(benzyloxy)-6-methoxyisoquinoline (**6**)

^{13}C NMR (125 MHz, $CDCl_3$)

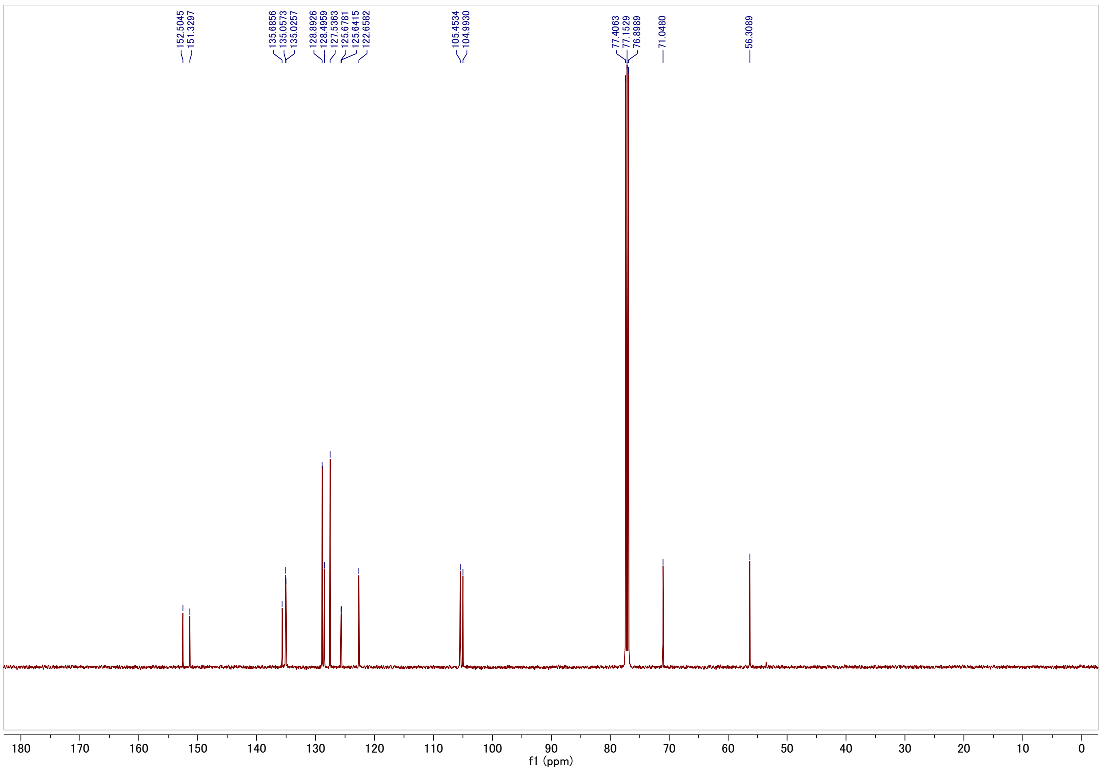

¹H NMR (500 MHz, $CDCl_3$)

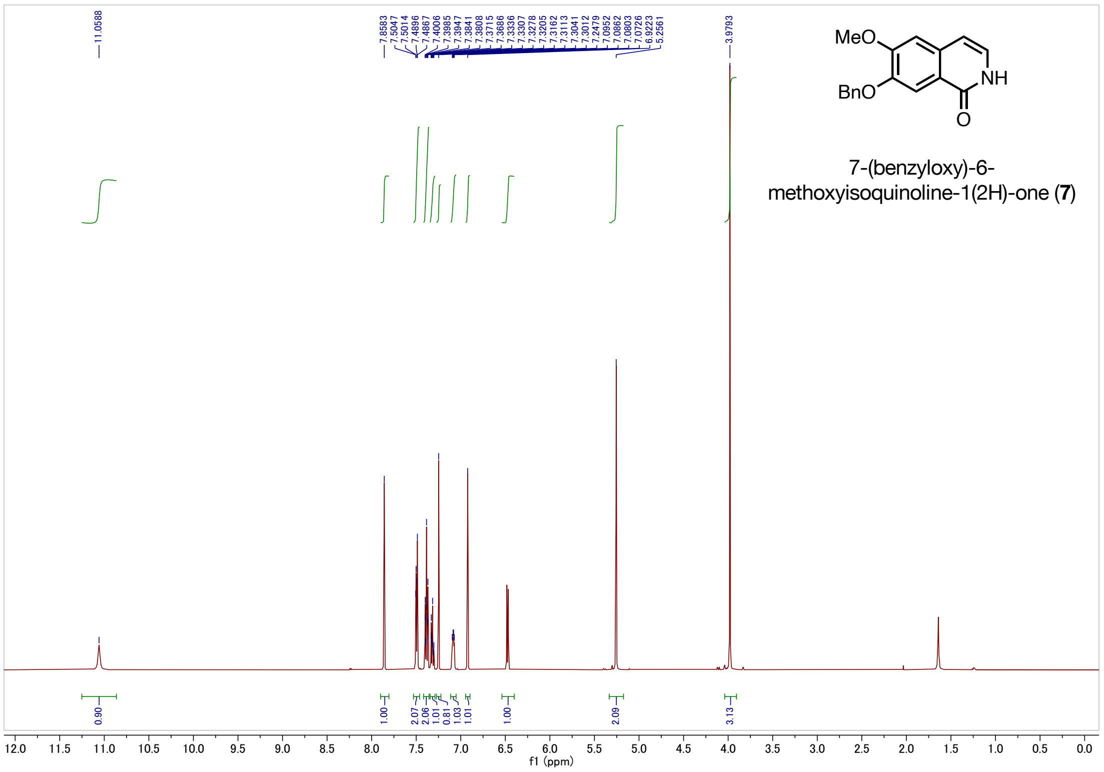

¹³C NMR (125 MHz, $CDCl_3$)

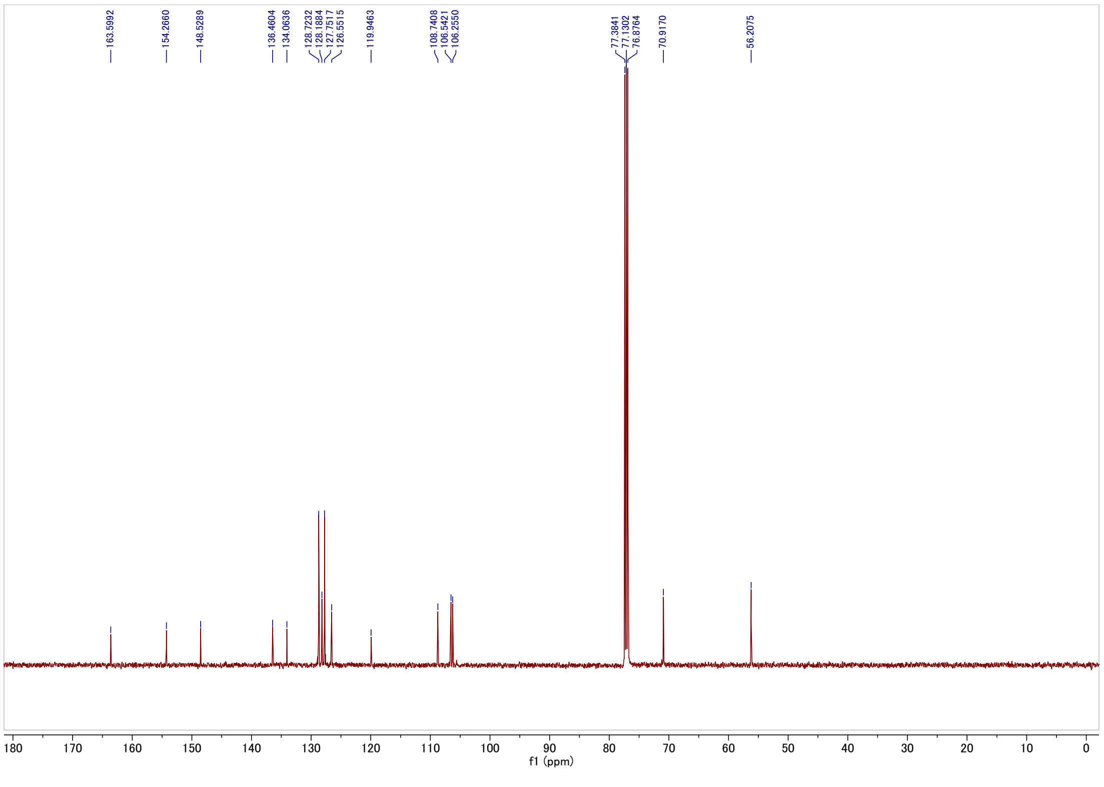

^{1}**H NMR** (500 MHz, $CDCl_3$)

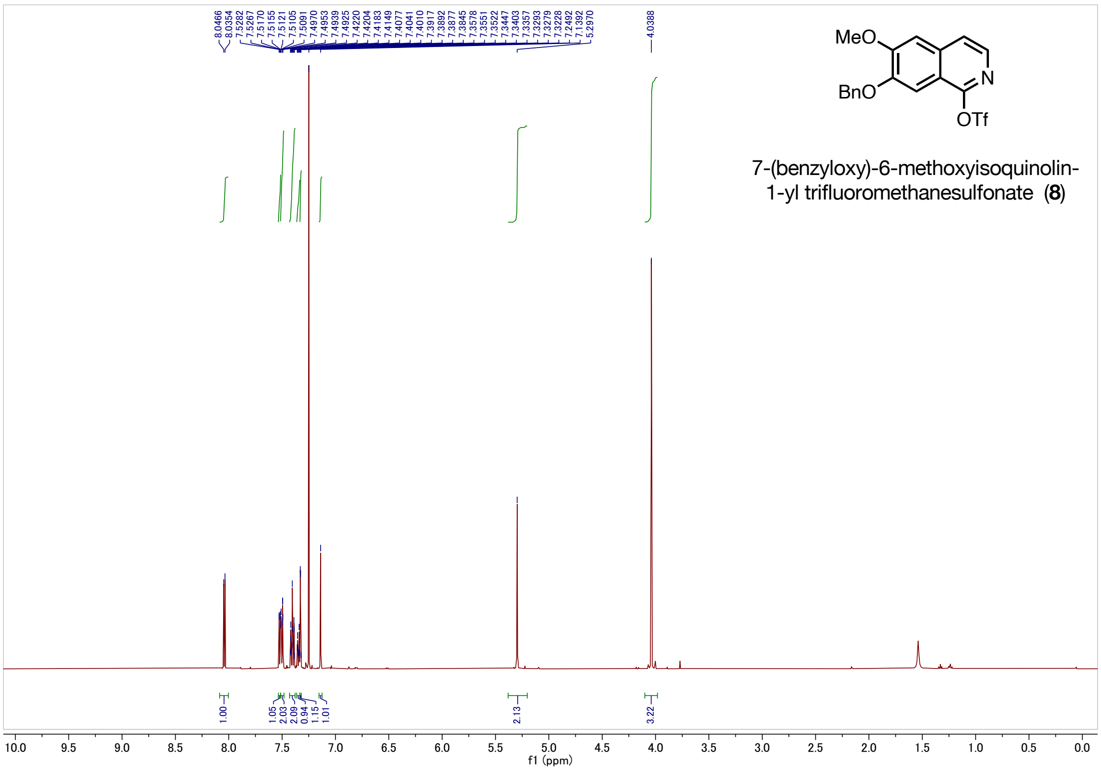

^{13}**C NMR** (125 MHz, $CDCl_3$)

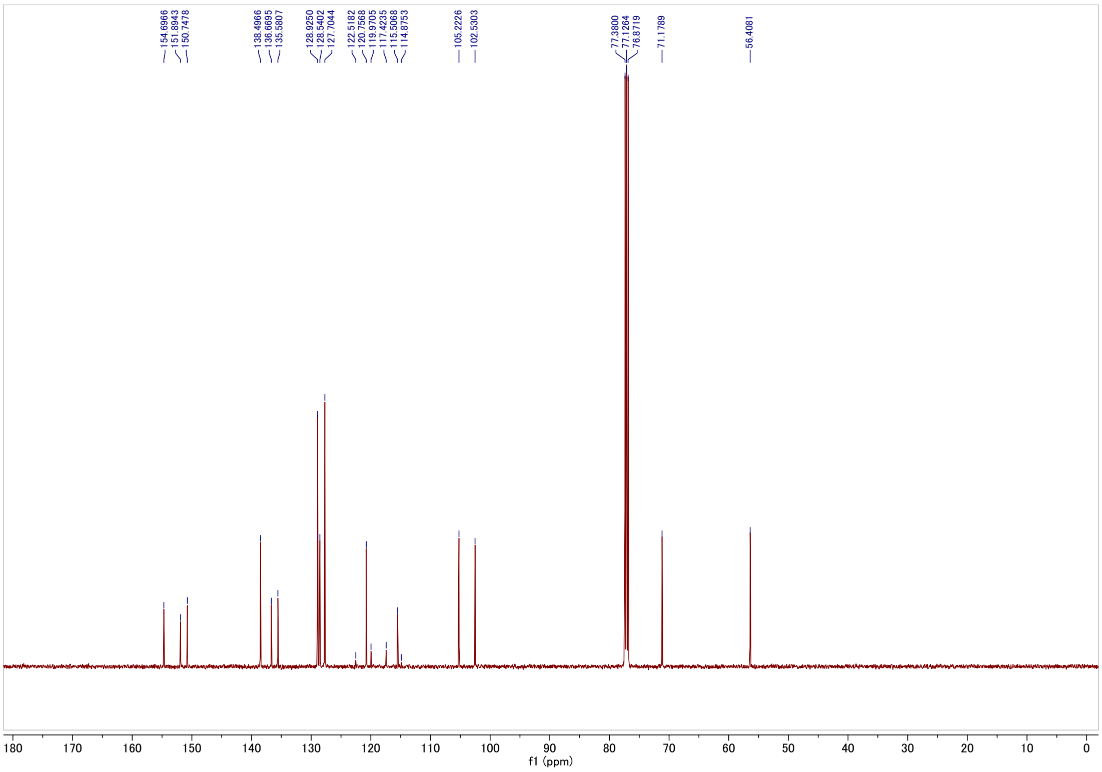

^{1}H NMR (500 MHz, $CDCl_3$)

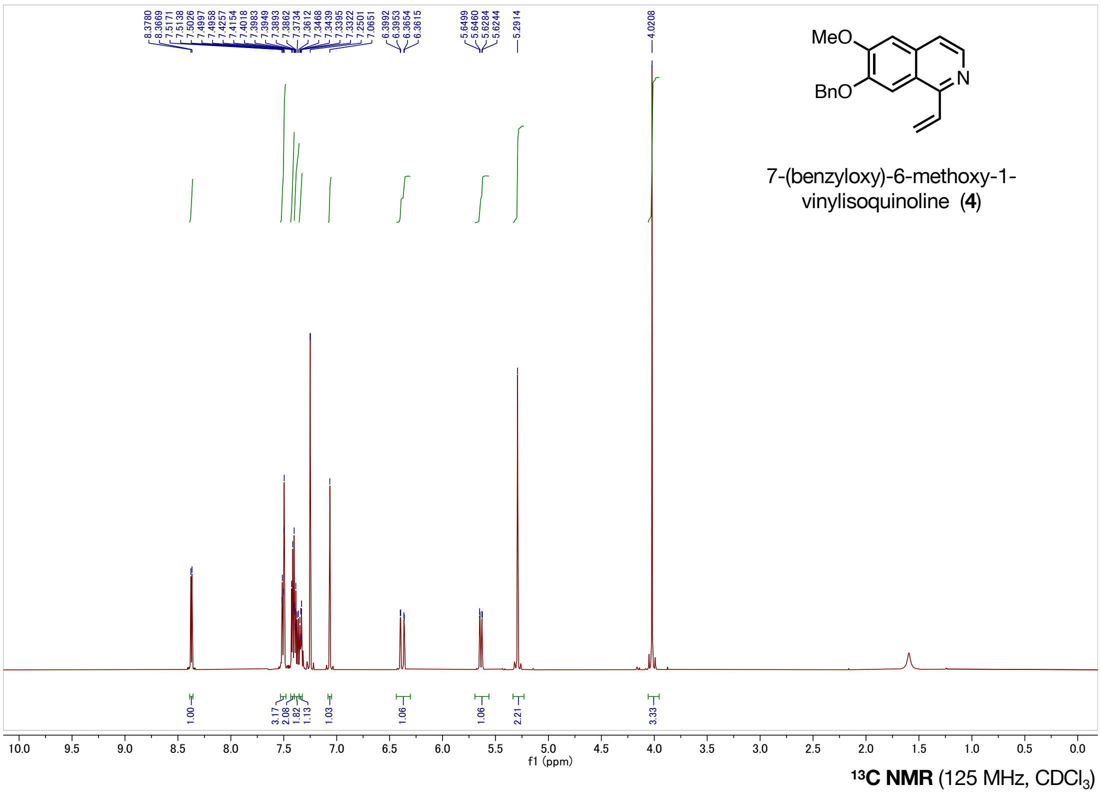

^{13}C NMR (125 MHz, $CDCl_3$)

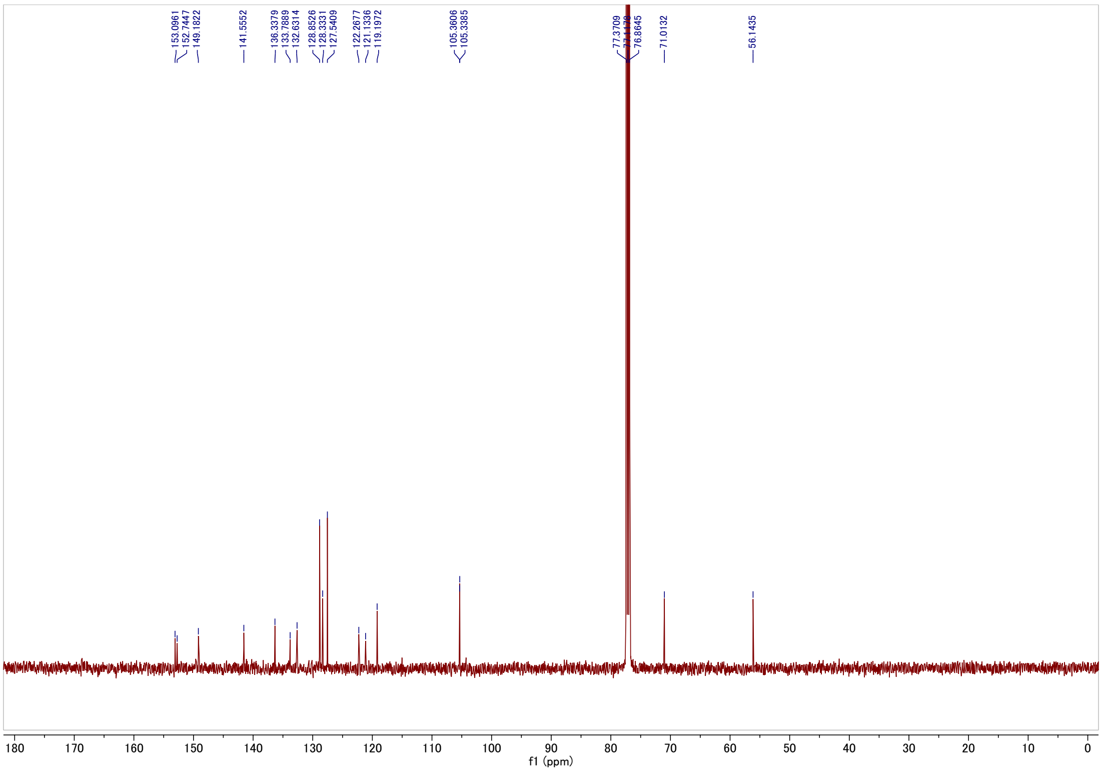

¹H NMR (500 MHz, $CDCl_3$)

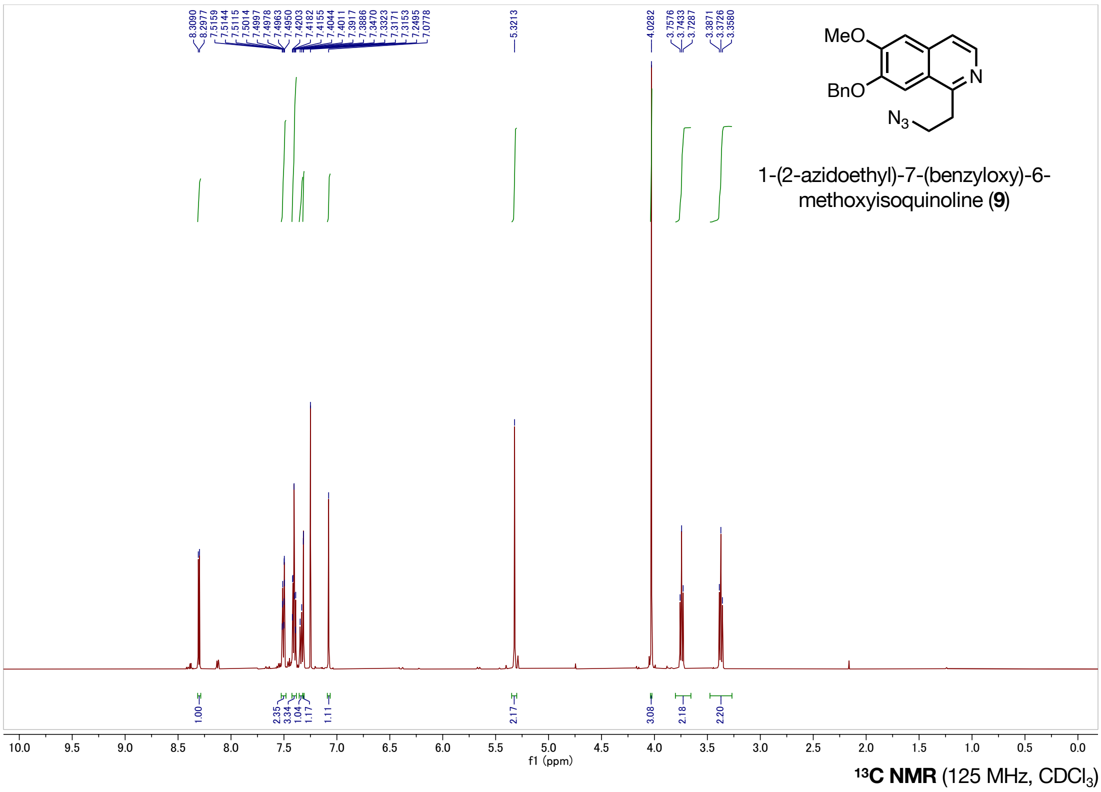

1-(2-azidoethyl)-7-(benzyloxy)-6-methoxyisoquinoline (**9**)

¹³C NMR (125 MHz, $CDCl_3$)

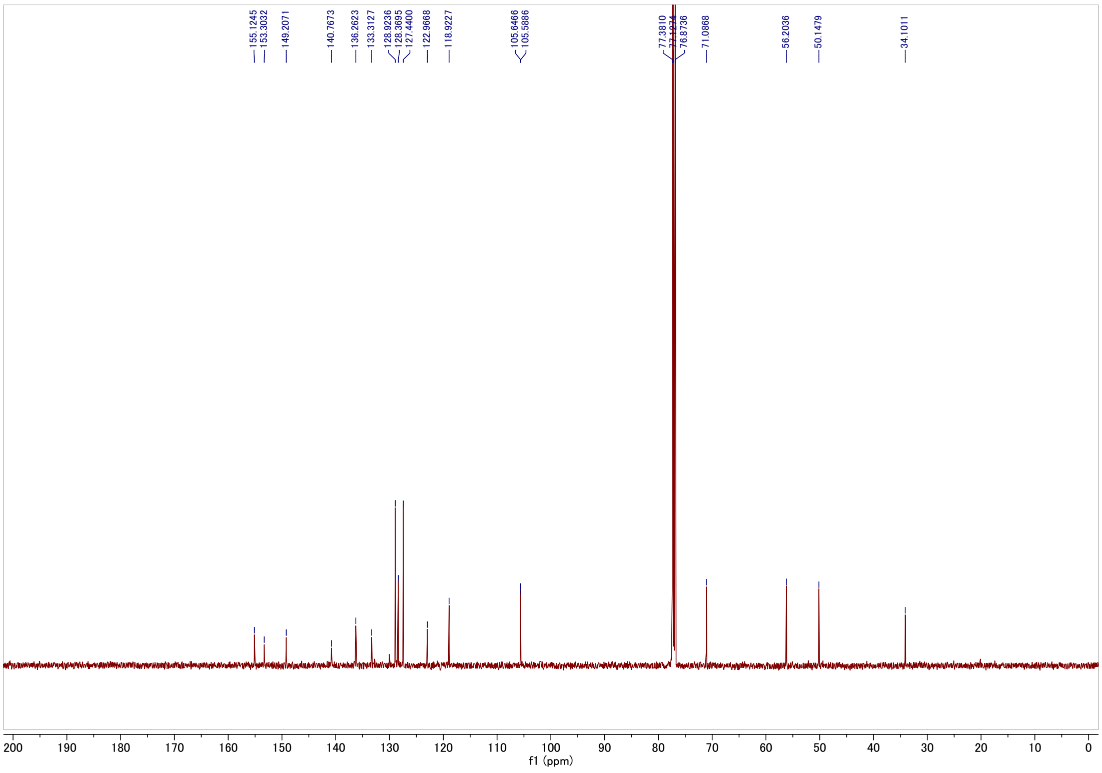

^{1}H NMR (500 MHz, $CDCl_3$)

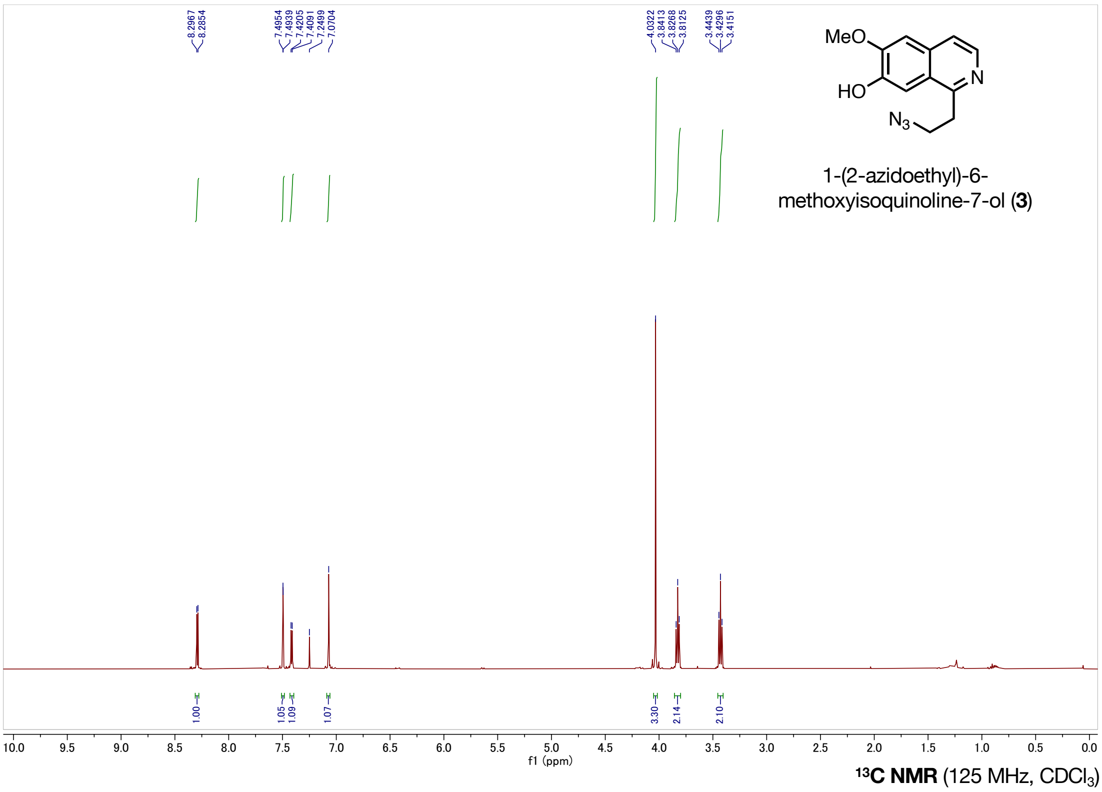

^{13}C NMR (125 MHz, $CDCl_3$)

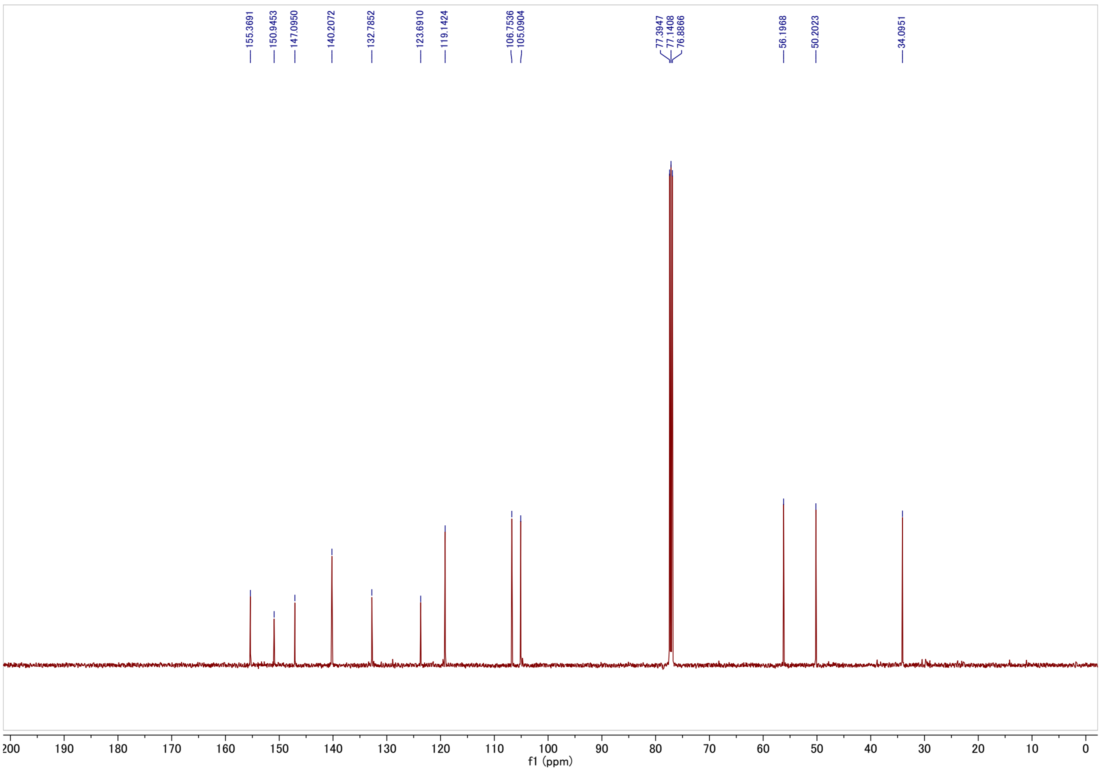

¹H NMR (500 MHz, $CDCl_3$)

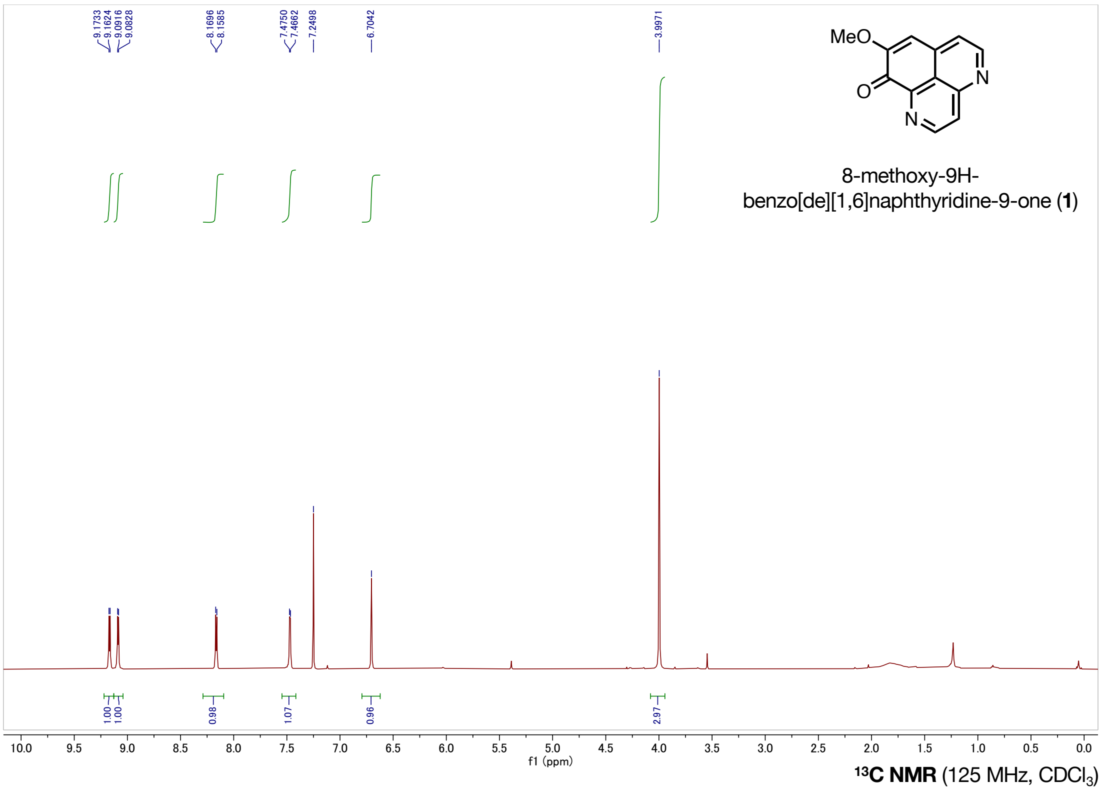

¹³C NMR (125 MHz, $CDCl_3$)

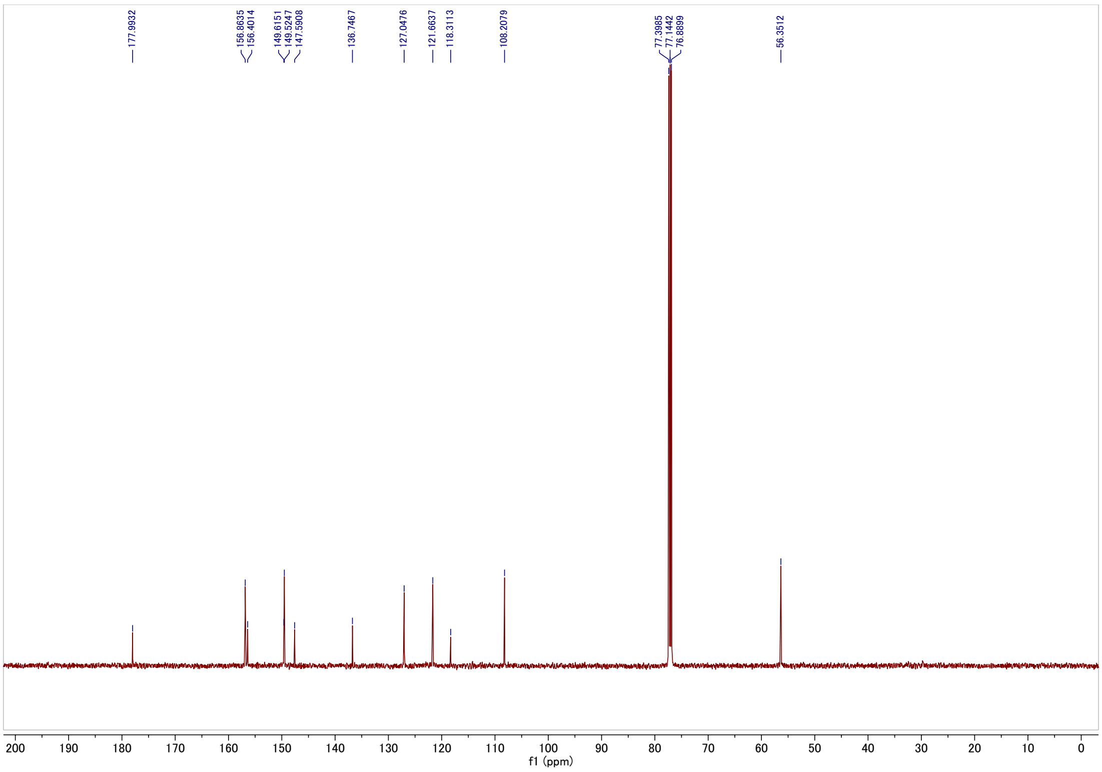

1H NMR (500 MHz, $CDCl_3$)

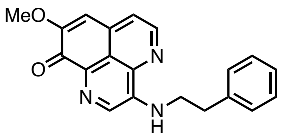

8-methoxy-3-(phenethylamino)-9H-benzo[de][1,6]naphthyridine-9-one (**2**)

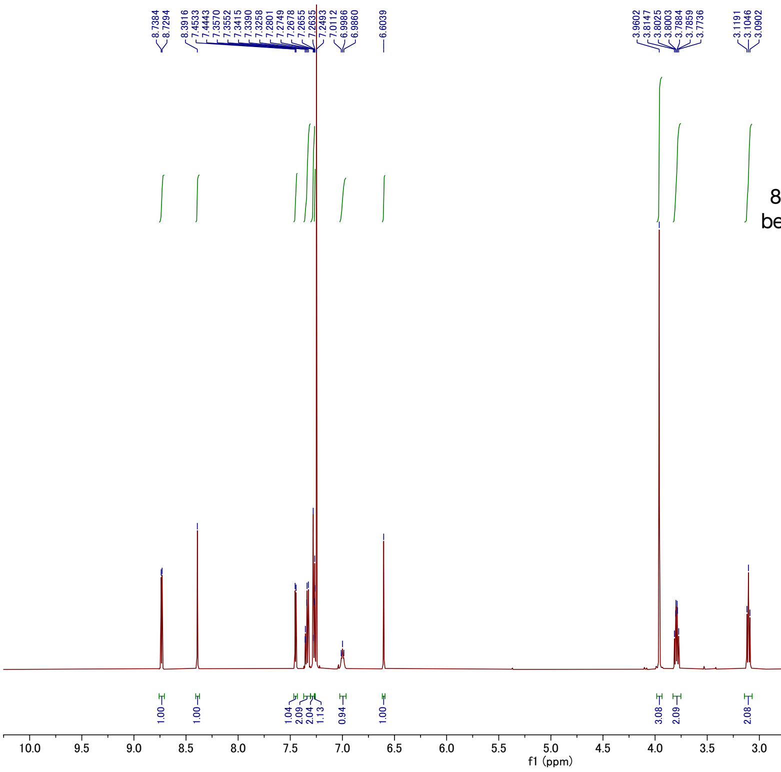

13C NMR (125 MHz, $CDCl_3$)

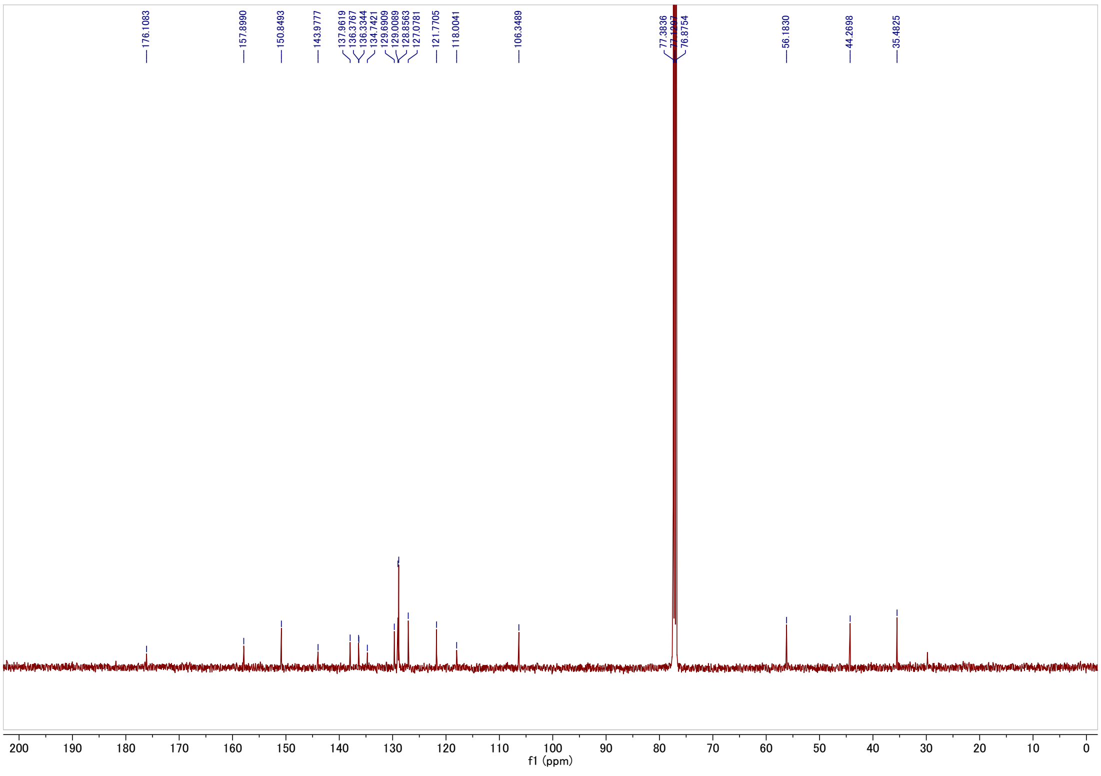

^{1}H NMR (500 MHz, $CDCl_3$)

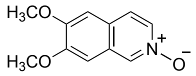

6,7-dimethoxyisoquinoline 2-oxide (**11**)

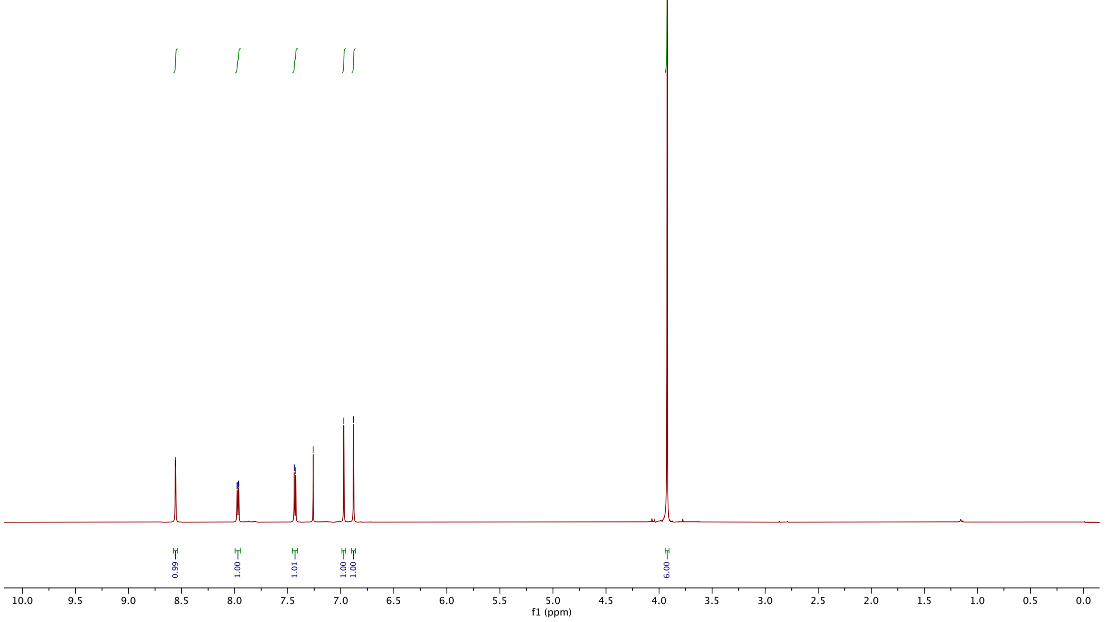

^{13}C NMR (125 MHz, $CDCl_3$)

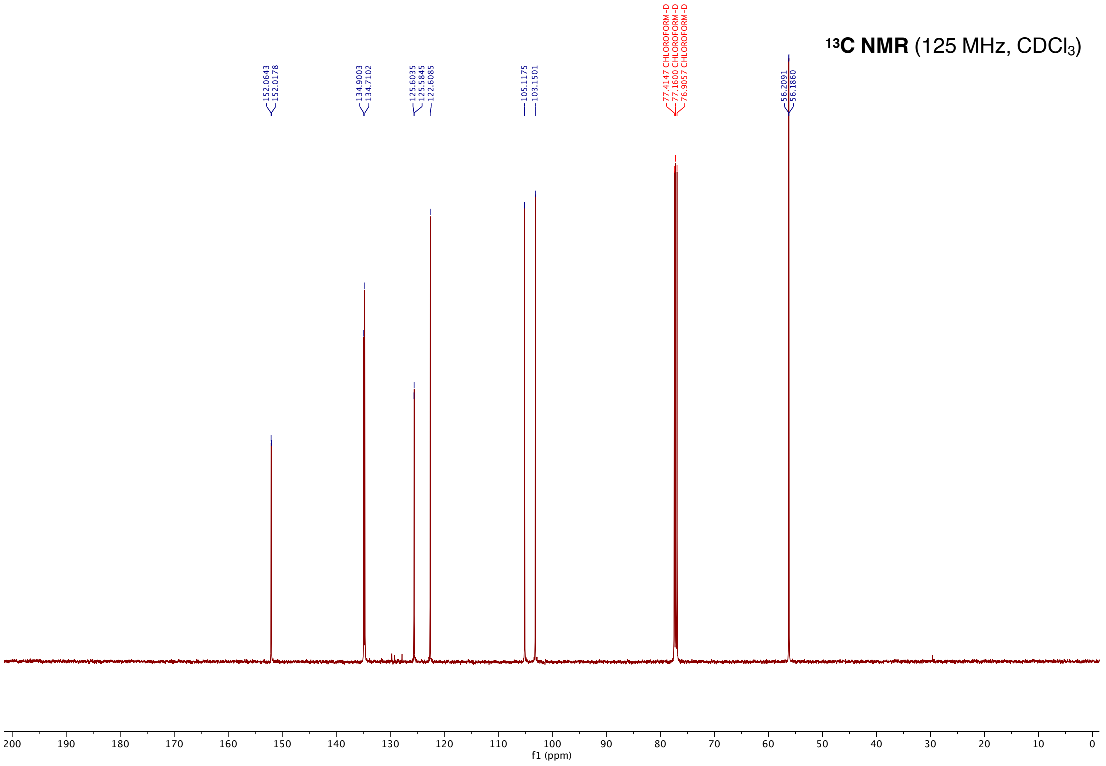

^{1}H NMR (500 MHz, $CDCl_3$)

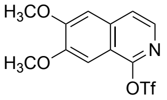

6,7-dimethoxyisoquinolin-1-yl trifluoromethanesulfonate (**13**)

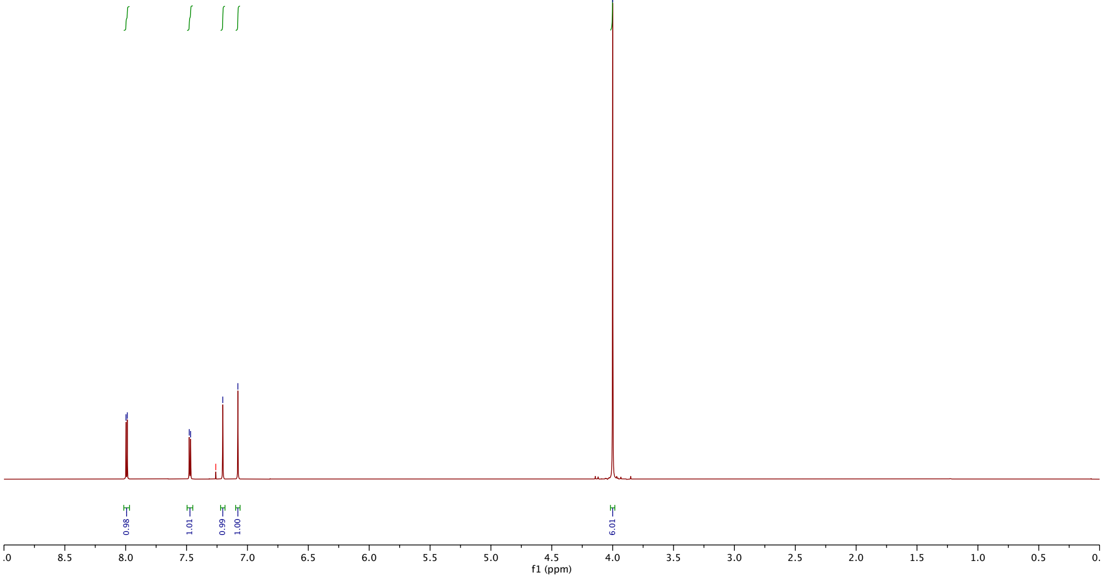

^{13}C NMR (125 MHz, $CDCl_3$)

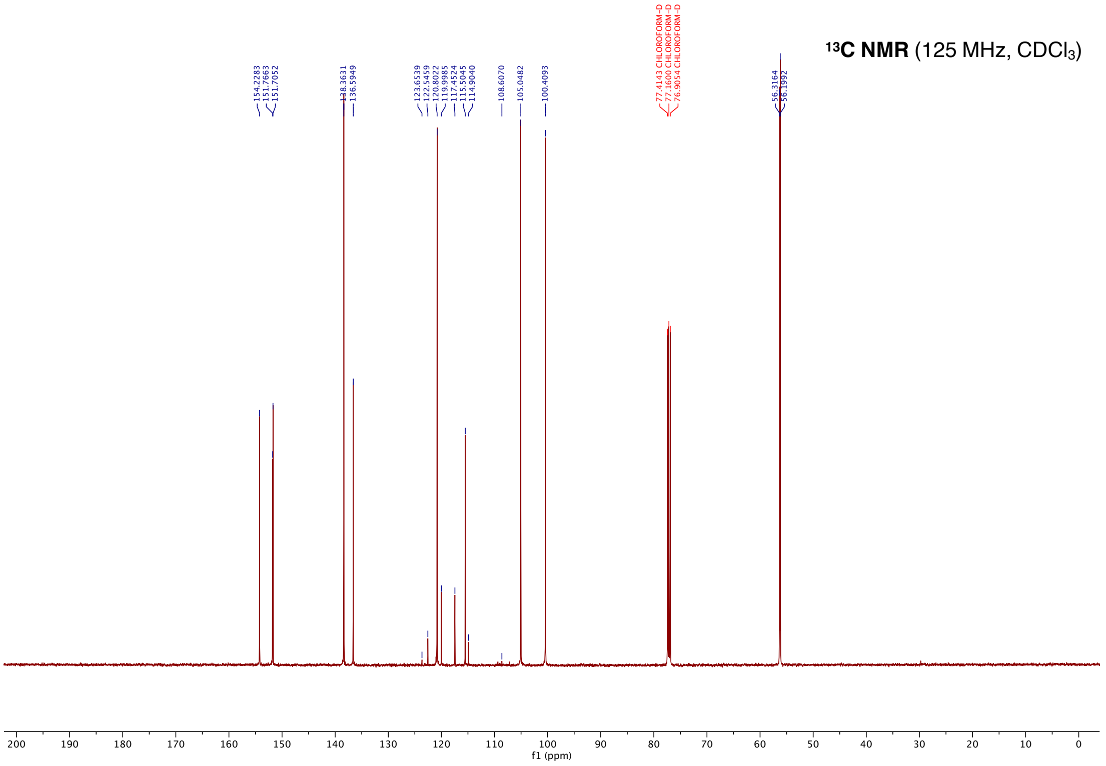

¹H NMR (500 MHz, $CDCl_3$)

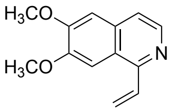

6,7-dimethoxy-1-vinylisoquinoline (**14**)

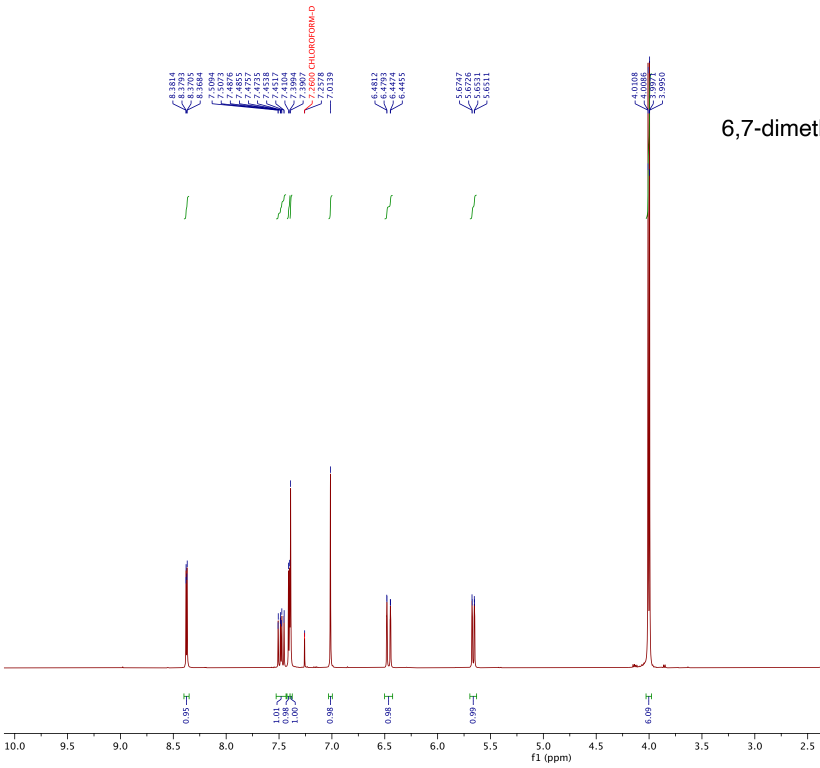

¹³C NMR (125 MHz, $CDCl_3$)

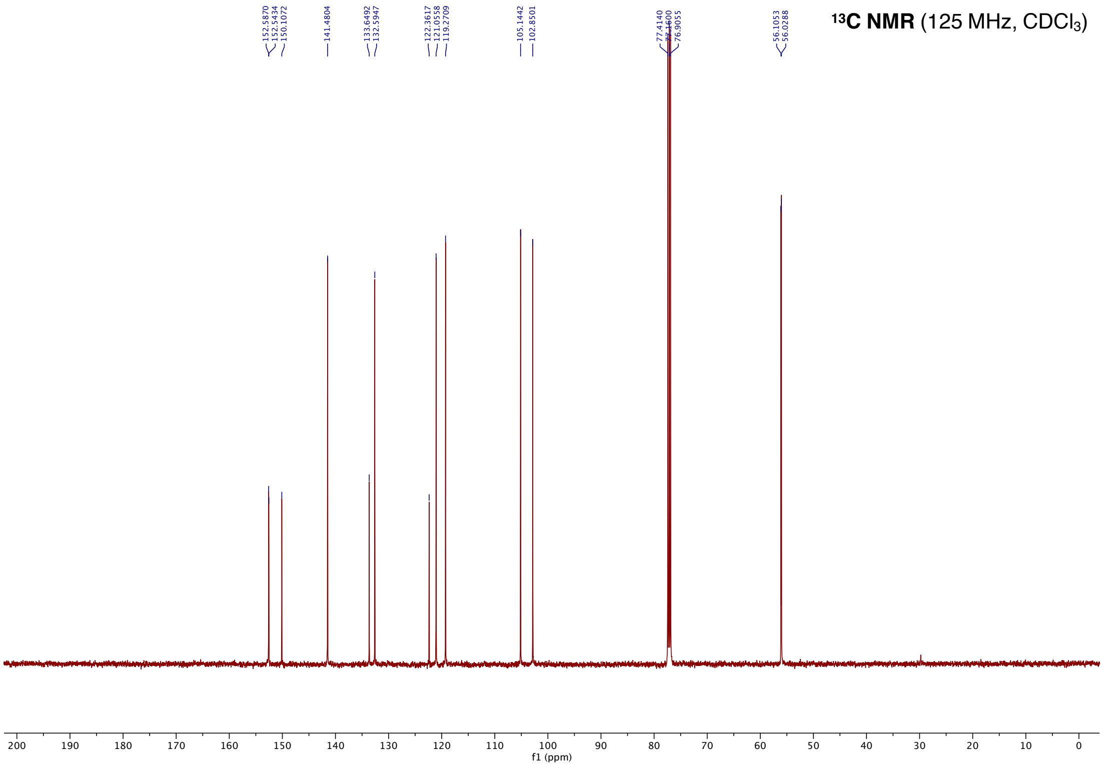

¹H NMR (500 MHz, $CDCl_3$)

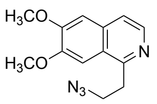

1-(2-azidoethyl)-6,7-dimethoxyisoquinoline (**15**)

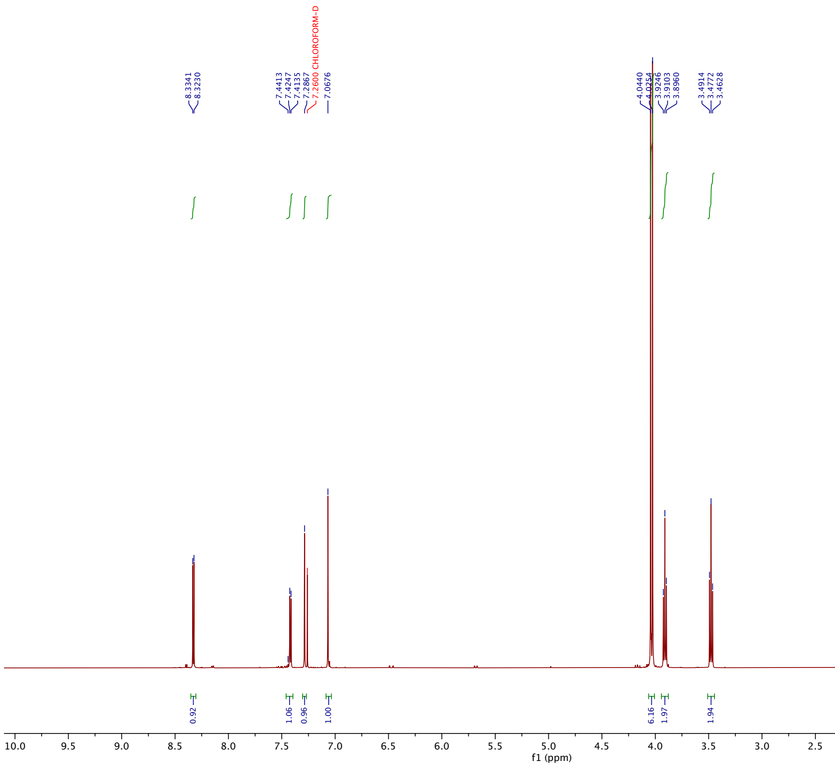

¹³C NMR (125 MHz, $CDCl_3$)

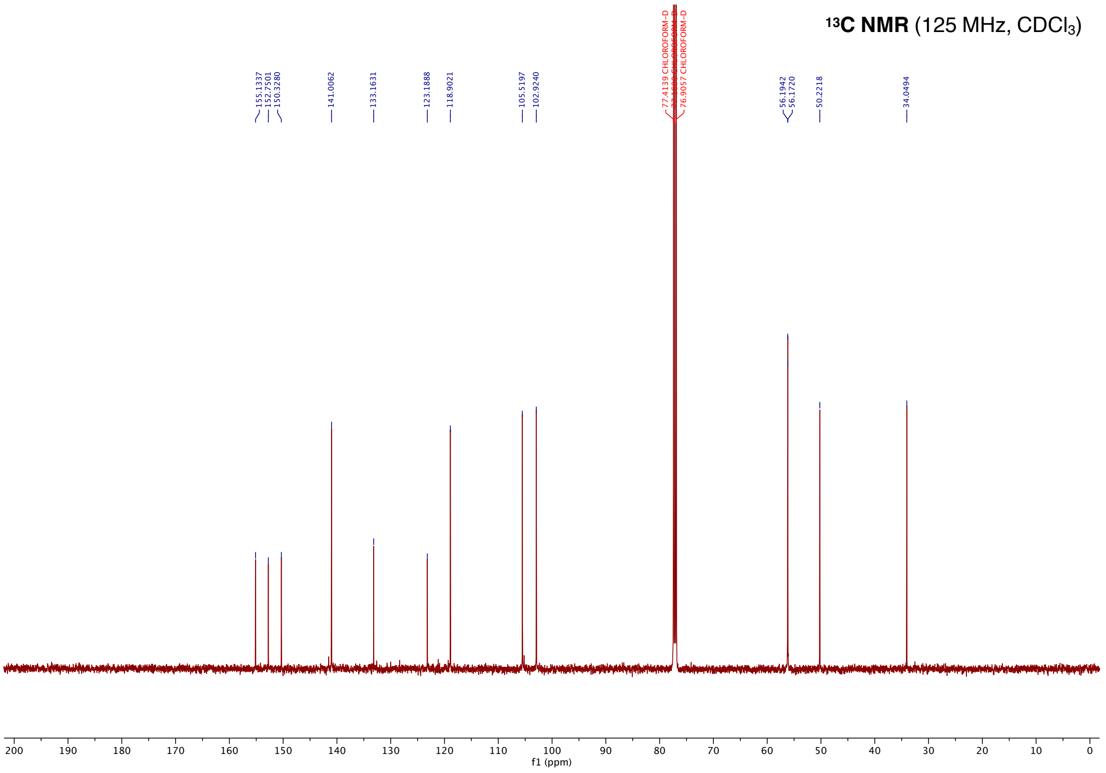

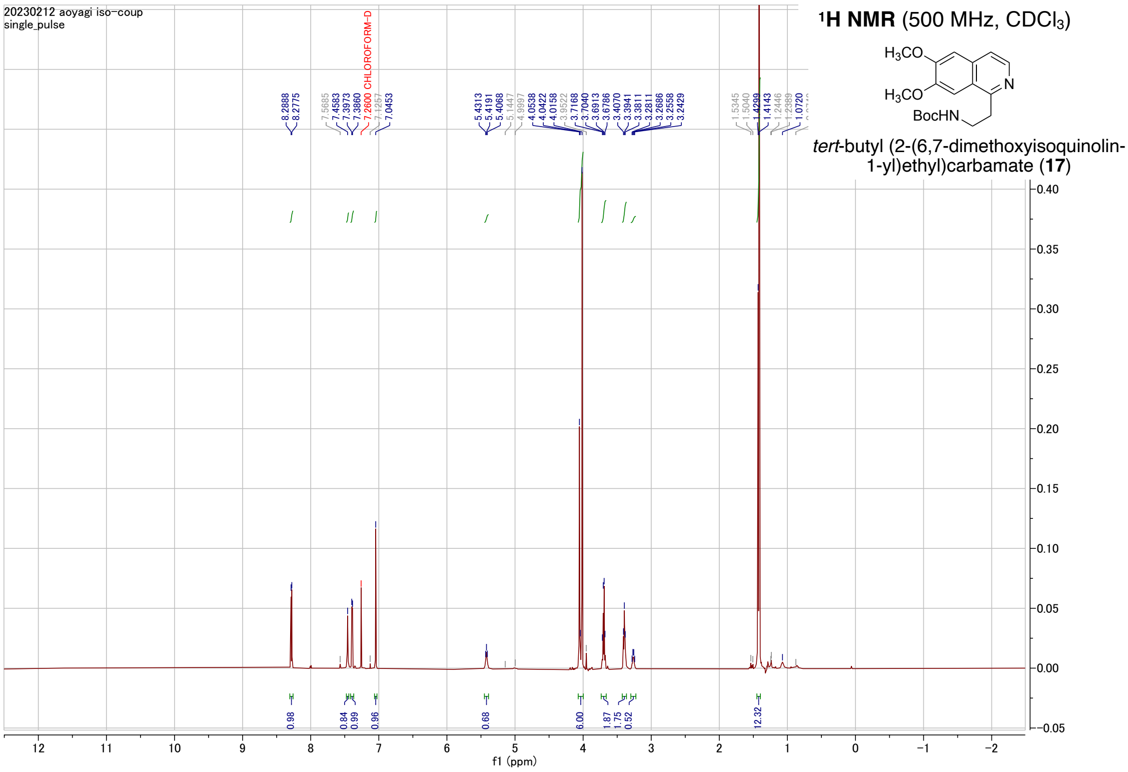

20230212 aoyagi iso-coup
single_pulse
1H NMR (500 MHz, CDCl3)
H3CO
H3CO
N
BocHN
tert-butyl (2-(6,7-dimethoxyisoquinolin-1-yl)ethyl)carbamate (17)
8.2888
8.2775
7.4583
7.3973
7.3860
7.2600 CHLOROFORM-D
7.0453
5.4313
5.4191
5.4068
4.0538
4.0422
4.0158
3.7168
3.7040
3.6913
3.6786
3.4070
3.3941
3.3811
3.2811
3.2686
3.2558
3.2429
1.4239
1.4143
1.0720
0.98
0.84
0.99
0.96
0.68
6.00
1.87
1.75
0.52
12.32
f1 (ppm)

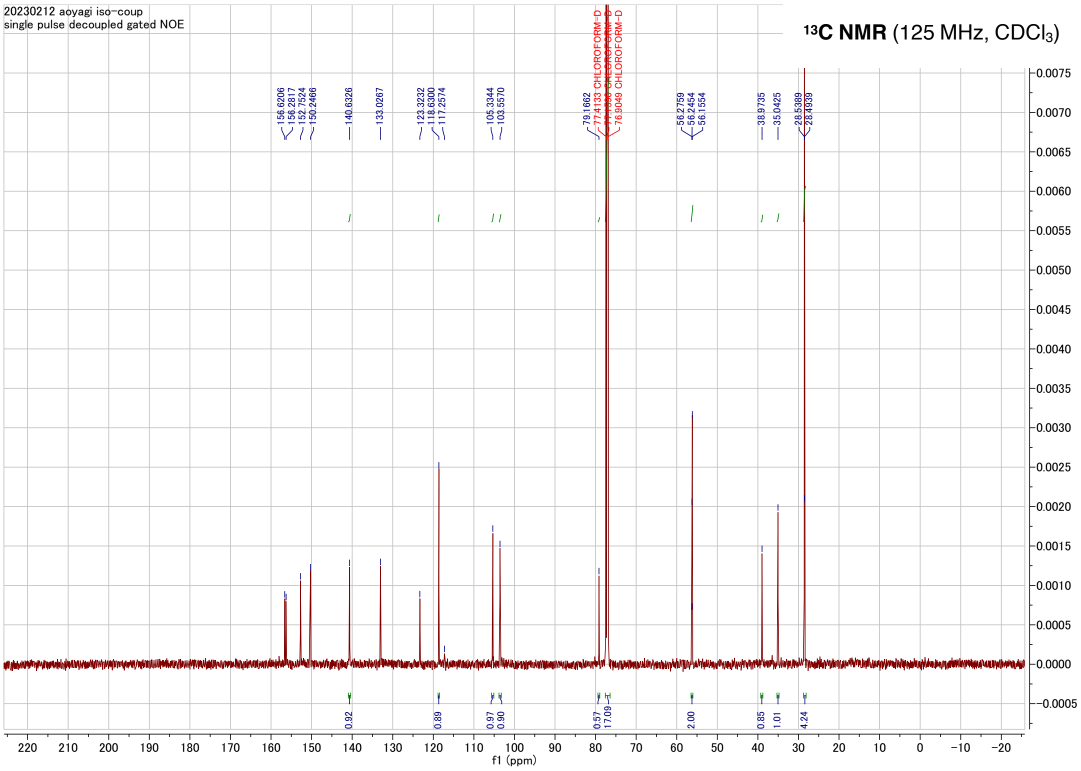

20230212 aoyagi iso-coup
single pulse decoupled gated NOE
13C NMR (125 MHz, CDCl3)
156.6206
156.2817
152.7524
150.2466
140.6326
133.0267
123.3232
118.6300
117.2574
105.3344
103.5570
79.1662
77.4133 CHLOROFORM-D
76.9049 CHLOROFORM-D
56.2759
56.2454
56.1554
38.9735
35.0425
28.5389
28.4939
0.92
0.89
0.97
0.90
0.57
17.09
2.00
0.85
1.01
4.24
f1 (ppm)
